# Blocking the TRAIL-DR5 Pathway Reduces Cardiac Ischemia–Reperfusion Injury by Decreasing Neutrophil Infiltration and Neutrophil Extracellular Traps Formation

**DOI:** 10.1007/s10557-024-07591-z

**Published:** 2024-06-20

**Authors:** Xuance Wang, Ran Xie, Dan Zhao, Guiling Wang, Lijie Zhang, Wei Shi, Yanyan Chen, Tingting Mo, Yuxin Du, Xuefei Tian, Wanjun Wang, Run Cao, Yuanfang Ma, Yinxiang Wei, Yaohui Wang

**Affiliations:** 1 Joint National Laboratory for Antibody Drug Engineering, Henan University, Kaifeng, 475004 P.R. China; 2 The College of Medical Technology, Shangqiu Medical College, Shangqiu, 476000 P.R. China; 3 The First Affiliated Hospital, Henan University, Kaifeng, 475004 P.R. China

**Keywords:** Cardiac ischemia–reperfusion, TRAIL, DR5, NETs, PAD4

## Abstract

**Purpose:**

Acute myocardial infarction (AMI) is a leading cause of mortality. Neutrophils penetrate injured heart tissue during AMI or ischemia–reperfusion (I/R) injury and produce inflammatory factors, chemokines, and extracellular traps that exacerbate heart injury. Inhibition of the TRAIL-DR5 pathway has been demonstrated to alleviate cardiac ischemia–reperfusion injury in a leukocyte-dependent manner. However, it remains unknown whether TRAIL-DR5 signaling is involved in regulating neutrophil extracellular traps (NETs) release.

**Methods:**

This study used various models to examine the effects of activating the TRAIL-DR5 pathway with soluble mouse TRAIL protein and inhibiting the TRAIL-DR5 signaling pathway using DR5 knockout mice or mDR5-Fc fusion protein on NETs formation and cardiac injury. The models used included a co-culture model involving bone marrow-derived neutrophils and primary cardiomyocytes and a model of myocardial I/R in mice.

**Results:**

NETs formation is suppressed by TRAIL-DR5 signaling pathway inhibition, which can lessen cardiac I/R injury. This intervention reduces the release of adhesion molecules and chemokines, resulting in decreased neutrophil infiltration and inhibiting NETs production by downregulating PAD4 in neutrophils.

**Conclusion:**

This work clarifies how the TRAIL-DR5 signaling pathway regulates the neutrophil response during myocardial I/R damage, thereby providing a scientific basis for therapeutic intervention targeting the TRAIL-DR5 signaling pathway in myocardial infarction.

## Introduction

Acute myocardial infarction is now the primary cause of death from cardiovascular illness, which poses a serious hazard to human health due to its high prevalence [1, 2]. Current treatment options for acute myocardial infarction include thrombolysis, bypass grafting, and percutaneous coronary intervention (PCI) [3, 4]. However, these interventions may induce cardiac I/R injury [5]. Therefore, the development of effective therapeutic procedures that may overcome the shortcomings of present treatment modalities is crucial.

Cardiac I/R injury is a complex process wherein neutrophils recruit more inflammatory cells and release pathogenic proteases, including matrix metalloproteinases, chemotactic factors, and myeloperoxidase (MPO), which exacerbate cardiac injury in the infarcted myocardium [6, 7]. Furthermore, it has been demonstrated that NETs formation, characterized by the release of web-like structures during neutrophil cell death, also contributes to the inflammatory damage process in myocardial infarction [8, 9]. NETs are complex fibrous structures consisting of chromatin DNA derived from neutrophils and a diverse range of intracellular granular proteins, which play a crucial role in capturing and eliminating pathogens, thereby exerting potent anti-infective effects [10]. However, NETs can promote platelet adhesion and aggregation, providing a supportive structure for thrombus formation [11]. They also provoke acute myocardial infarction by increasing fibrin deposition and enhancing red blood cell agglutination, which causes thrombus development [12, 13]. These results imply that NETs are essential to the pathophysiology of myocardial I/R injury and that treatments that specifically target NETs may be effective.

TRAIL and its receptor DR5 are essential components of the pathophysiology of myocardial infarction, including the induction of cardiomyocyte apoptosis and the coordination of immune cell recruitment and activation, both of which lead to cardiac damage [14–16]. However, it remains unclear whether TRAIL can induce the release of NETs to exacerbate cardiac I/R injury. We used mDR5-Fc protein and DR5 knockout mice to further understand the regulatory function and mechanism of the TRAIL-DR5 signaling pathway in NET production following cardiac IR injury.

## Materials and Methods

### Animals

The C57BL/6N male mice, obtained from Vital River Company, were acclimated to the environment for one week prior to the study. The DR5 Knockout mice were generously provided by Professor Yinming Liang (Xinxiang Medical University), and bred in the animal facility of Joint National Laboratory for Antibody Drug Engineering. Mice were housed in a specific pathogen-free (SPF) environment, with up to five animals per cage, following a 12-h light/dark cycle, maintaining relative humidity between 55–70%, temperature conditions at 22–25 °C, and provided ad libitum access to food and water.

For the mice cardiac I/R injury model, anesthesia was induced using 2% isoflurane while ensuring proper disinfection with iodine solution and alcohol. A thoracic incision between the ribs allowed for exposure of the heart. Ligation of the left anterior descending coronary artery (LAD) was accomplished utilizing a 6–0 suture for a period lasting 40 min, after which reperfusion commenced upon release of this ligation. Subsequently, muscle and skin were sutured using a 4–0 suture for closure.

For Evan’s blue and TTC double staining, mice were killed 24 h after I/R injury. The blood vessels at the ligation site were ligated using a 4–0 suture and followed by Evans blue injection through the aorta. The hearts were excised and cryopreserved at -80 °C. The hearts under the ligation site were cut into five slices for TTC staining, and quantification of the infarct (white), risk (red), and non-infarct (blue) areas were analyzed using ImageJ software.

### Preparation of Mouse DR5-Fc Fusion Protein

The extracellular segment of mouse DR5 and the Fc region of mouse IgG1 were constructed into the pcDNA3.1 vector and subsequently transfected into 293F cells. The mDR5-Fc protein was purified from 293F culture supernatant using protein A affinity chromatography, followed by buffer exchange into PBS. Subsequently, the purified protein was filtered through a 0.22 μm membrane and quantified using the BCA assay kit. Purity assessment was performed via SDS-PAGE Coomassie Brilliant Blue staining.

### Neutrophil Depletion

To deplete neutrophils in mice, an anti-Ly6G antibody (Biolegend, 127,649) or isotype antibody (Biolegend, 400,565) was administered i.v. 0.2 mg per mouse, and the depletion efficiency was assessed using flow cytometry.

### Isolation and Culture of Primary Cells

For isolation of neutrophils from mouse bone marrow, mice were euthanized by cervical dislocation. The bone marrow was flushed into 1640 medium and filtered through a 40 μm sieve. Subsequently, cells were centrifuged at room temperature for 5 min at 500 g and resuspended in 2 mL of supplemented 1640 medium containing 10% FBS. The cell suspension was then transferred to a new 15 mL centrifuge tube containing Percoll solutions with concentrations of 78% and 65%. Centrifugation was performed at a speed of 2500 rpm for 30 min. The cell layer located at the interface between the layers of Percoll solutions was carefully collected into another clean 15 mL centrifuge tube, followed by washing with PBS. Finally, purified neutrophils were obtained and cultured short-term in a complete growth medium containing 10% FBS supplemented with 1640 medium.

The isolation of cardiomyocytes was performed according to a previously described protocol [14]. Briefly, neonatal rat ventricular myocytes (NRVMs) were isolated from 2 d old Wistar rats by mincing the hearts and digesting in a C-tube containing enzymatic solution (Worthington Biochemical) using gentleMACS™ (Miltenyi Biotec). Cardiomyocytes were then collected through percoll centrifugation and cultured in a medium supplemented with 20% FBS (Gibco).

### Co-Culture of Neutrophil Derived NETs and NRVM

Mouse bone marrow-derived neutrophils were stimulated with mTRAIL alone or in combination with mDR5-Fc, washed twice with culture medium, resuspended in 1640 medium supplemented with 10% FBS, and subsequently co-cultured with NRVM cells. After a duration of 24 h, the supernatant of the NRVM cell culture was collected to quantify LDH levels, while NRVM apoptosis was assessed using a TUNEL assay.

### Immunohistochemical

The left and right atria appendages were incised, and the left ventricle and right ventricle were flushed with 15–20 mL of heparinized PBS thoroughly to remove any residual blood. Subsequently, the hearts were fixed in a solution containing 4% paraformaldehyde.

After embedding, sectioning, dewaxing, and hydration of the tissue, endogenous peroxidase activity was blocked using hydrogen peroxide. The tissue slices were incubated 1 h with 5% BSA at room temperature and then overnight for primary antibody binding at 4 °C. Secondary antibodies were then incubated at room temperature for 1 h. Finally, the slices were analyzed using a microscope scanning system after chromogenic staining, hematoxylin counterstaining of nuclei, and sealing with a neutral resin.

### Immunofluorescence

The tissue sections underwent deparaffinization, hydration, and antigen retrieval and were subsequently incubated in 1% Triton X-100 PBS. Following blocking with 5% BSA, the slices were then incubated overnight at 4 °C with the primary antibody. Subsequently, the secondary antibody was applied, and cellular nuclei were stained with DAPI. Finally, fluorescence microscopy was employed to capture images of the slides.

For TUNEL staining, the heart tissue sections were subjected to a TUNEL staining kit according to the manufacturer’s protocol and subsequently examined using fluorescence microscopy.

### Western Blot

The mouse heart was lysed using RIPA lysis buffer supplemented with protease inhibitors and PMSF, followed by tissue homogenization. Subsequently, the lysate was incubated on ice for 30 min. After centrifugation, the supernatant was collected and subjected to BCA protein quantification. SDS-PAGE analysis was conducted, and the proteins were transferred onto a PVDF membrane. The membrane was then incubated with 5% skim milk and primary antibody overnight at 4 °C. The membrane was incubated with a secondary antibody and substrate for detection.

### qPCR

RNA was extracted from mouse cardiac tissue using the TRIZOL method, followed by reverse transcription into cDNA. qPCR was performed and subsequent detection was carried out using QuantStudio 3. The primer sequences used for qPCR were as follows.

*Tnfa* AGGCGGTGCCTATGTCTCA, GGGAGGCCATTTGGGAACTTCT; *Il1b* TGCCACCTTTTGACAGTGATG, TGATGTGCTGCTGCGAGATT; *Mpo* AGTTGTGCTGAGCTGTATGGA, CGGCTGCTTGAAGTAAAACAGG; *Cxcl15* TGGGTGAAGGCTACTGTTGG, AGCTTCATTGCCGGTGGAAA; *Icam1* CCGCTACCATCACCGTGTATT, GGTGAGGTCCTTGCCTACTT; *Itgam* AAAGAACAACACACGCAGGC, CAGAACTGGTCGGAGGTTCC; *Pad4* GGCTCATTCCCCTCACCATC, TTGTCAGAAACCCTGCACAC; *Gapdh* GGTGAAGGTCGGTGTGAACG, CTCGCTCCTGGAAGATGGTG.

### Detection of Peripheral Blood and Plasma Samples

The quantification of plasma lactate dehydrogenase (LDH) and creatine kinase (CK) levels was conducted using biochemical instrumentation (IDEXX). For dsDNA detection, ELISA was performed according to the manufacturer’s protocol (Solarbio, P9740).

### Flow Cytometry

After perfusion with PBS, the mouse heart was dissected to remove adherent tissues and the aorta [17]. The mouse heart was then weighed, minced, and digested in a digestion solution containing type II collagenase (Worthington Biochemical), neutral protease (Roche), and DNase I (D4513, Sigma-Aldrich). Subsequently, the digestion was neutralized with 2%FBS, and the digested cells were passed through a 70 μm sieve and lysed red blood cells. Anti-mouse CD16/32 antibody (eBioscience, 14–0161-85) was used for blocking at 4 °C followed by incubation with antibodies (CD45, eBioscience, 47–0451-82; CD11b, eBioscience, 63–0112-82; Ly6G, eBioscience, 46–9668-82) at 4 °C in the dark for 30 min. Finally, flow cytometry analysis was performed using Bio-Rad ZE5.

### Quantification and Statistical Analysis

The data analysis in this study was performed using GraphPad Prism9. The results were presented as means ± SEM and subjected to statistical analysis using the unpaired *t* test or one-way ANOVA followed by Tukey’s post hoc test (*P < 0.05, **P < 0.01, ***P < 0.001, ****P < 0.001).

## Results

### Cardiac I/R Induced NETs Formation and Release

**Fig. 1:**
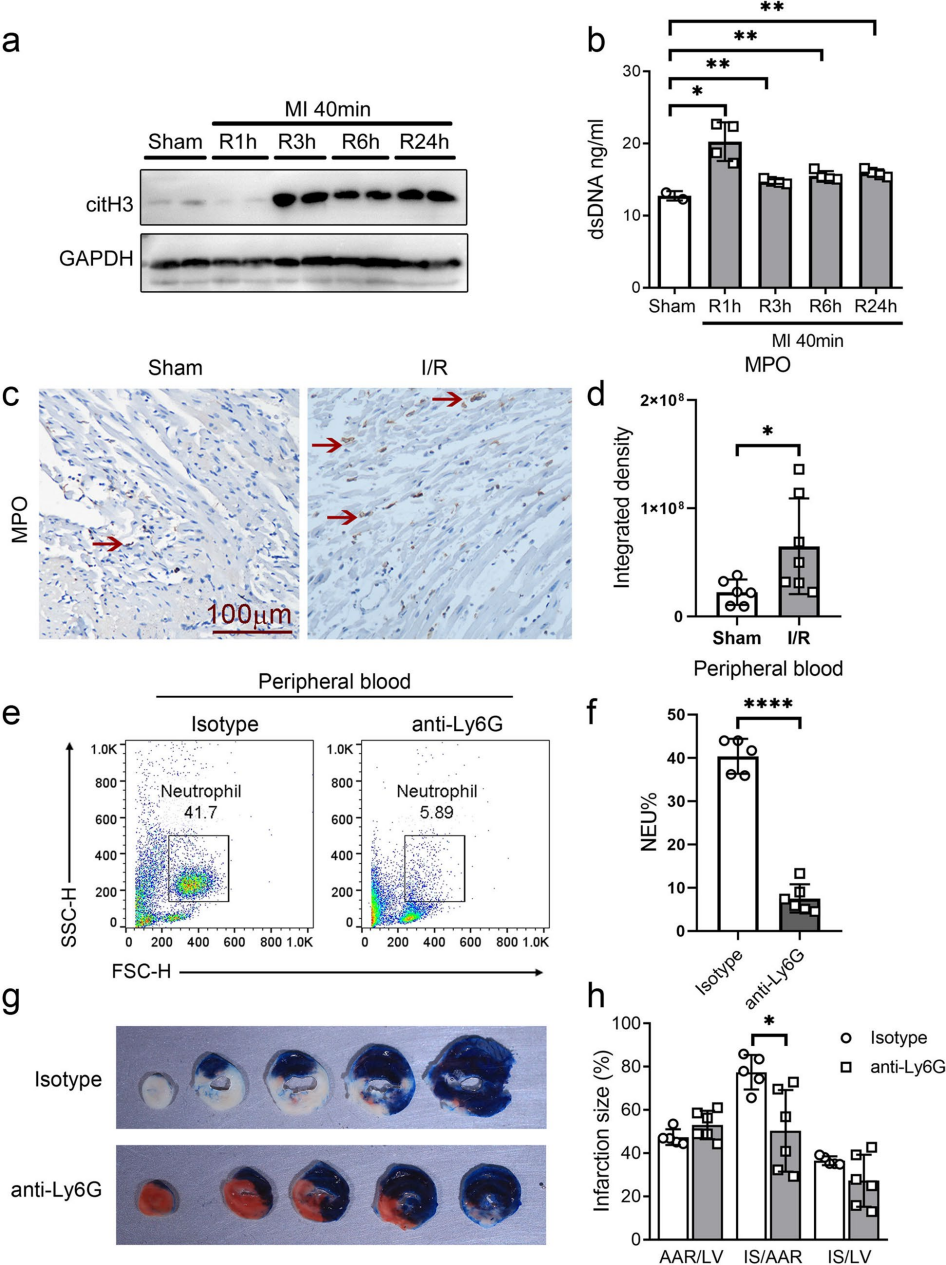
NETs were increased and involved in cardiac I/R injury. **a** The expression of citH3 in the hearts of mice was assessed by western blot analysis following sham surgery or 40 min of ischemia with subsequent reperfusion for the indicated periods. **b** Detection of dsDNA content in mouse plasma using ELISA assay following sham surgery or 40 min of ischemia with subsequent reperfusion for the indicated periods. **c–d** Representative IHC results (**c**) and statistical analysis (**d**) of MPO expression in mouse heart tissue following sham surgery or I40min/R24h. **e–f** Flow cytometry detection (**e**) and statistical analysis (**f**) of neutrophils clearance efficiency in the mice peripheral blood. **g–h** Representative results (**g**) and statistical (**h**) of TTC-Evans blue double stain heart sections after I40min/R24h treated with isotype or anti-Ly6G antibody for neutrophils depletion. AAR, area at risk (white, red); IS, infarction size (white); LV, left ventricle. Quantitative data are shown as means ± SEM. *P ≤ 0.05, **P ≤ 0.01, and ***P ≤ 0.001 as determined by a two-tailed *t* test (**b**, **d**, **f**, and **h**)

We looked at the release of NETs in the hearts of mice after I/R injury to determine whether NETs are involved in cardiac I/R injury. Citrullinated histone H3 (citH3), an essential component of NET formation reflecting NET production, exhibited increased expression at 3 and 6 h post-reperfusion, which remained elevated even at 24 h post-reperfusion as determined by Western blotting analysis (Fig. 1a). ELISA analysis revealed elevated levels of double-stranded DNA (dsDNA) content in the plasma following reperfusion compared to the sham group (Fig. 1b). MPO is one of the components present on the DNA scaffold of NETs. Therefore, we assessed the number of MPO-positive cells in the heart tissues at various time points after reperfusion and observed a significant increase in MPO-positive cells 24 h post-reperfusion compared to the sham group (Fig. 1c, d). Neutrophils undergo an inflammatory cell death mechanism called NETosis. To explore whether early-stage NETs contribute to impaired cardiac recovery after I/R injury, we employed Ly6G antibody for neutrophil depletion. Flow cytometry analysis confirmed the effective depletion of neutrophils (Fig. 1e, f), while TTC/Evans blue dual staining showed a reduction by 35% in myocardial infarct area upon neutralizing neutrophils compared to control group mice subjected to cardiac I/R injury (Fig. 1g, h). On the basis of these data, neutrophils may migrate into injured tissues during the initial phases of cardiac I/R injury, releasing extracellular traps that harm the heart.

### The Beneficial Effect of DR5 Knockout on Cardiac I/R Injury is Dependent on Neutrophil

**Fig. 2:**
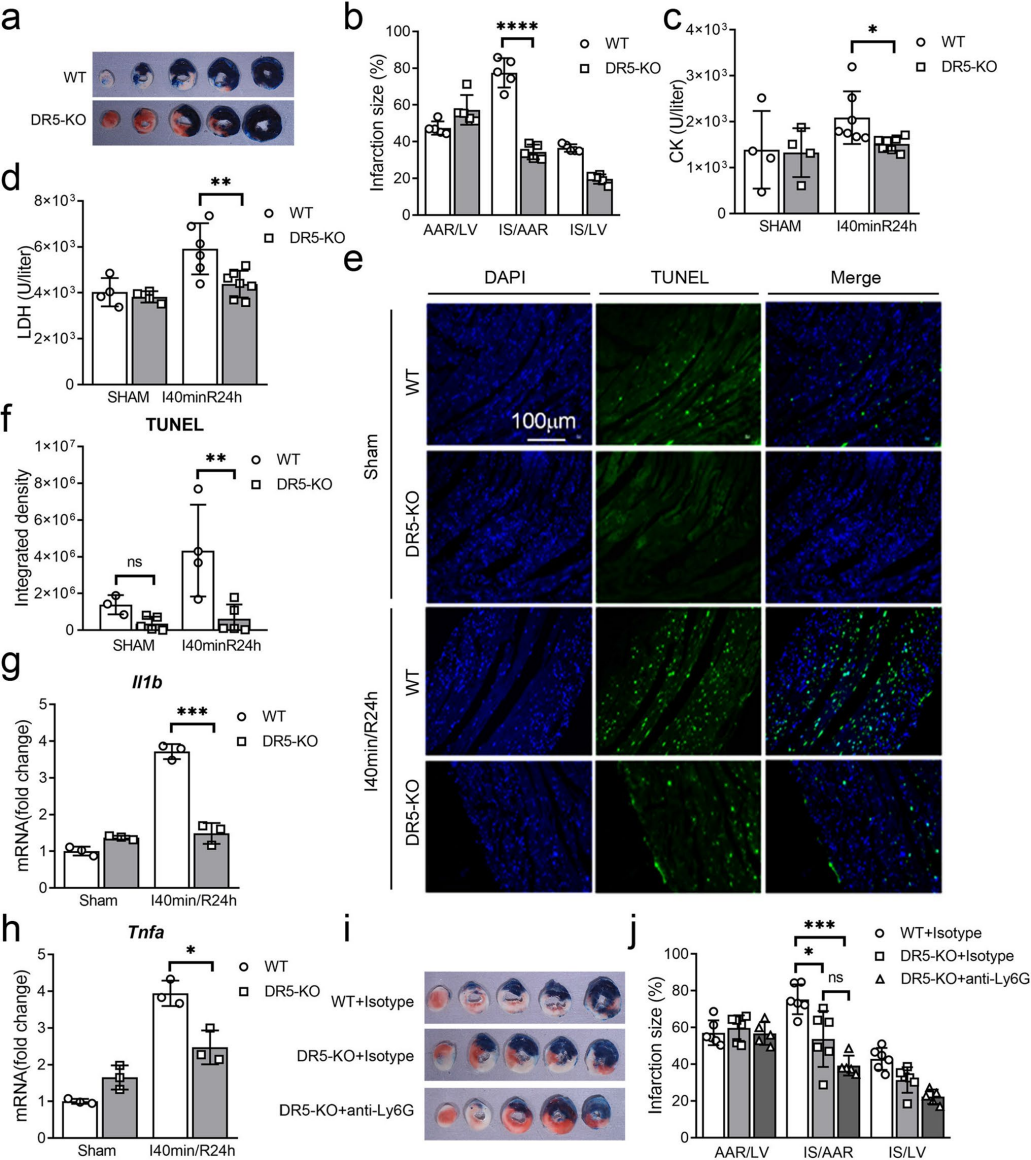
The protective effect against cardiac I/R injury in DR5 knockout mice relies on neutrophils. **a**–**b** Representative results (**a**) and statistical analysis (**b**) of TTC-Evans blue double stain heart sections following I40min/R24h in wild type (WT, *n* = 5) or DR5 knockout (DR5-KO, *n* = 5) mice. **c–d** Plasma concentrations of CK (**c**) and LDH (**d**) of WT or DR5-KO mice following sham or I 40 min/R 24 h were analyzed using the Catalyst Dx Chemistry Analyzer. **e–f** Immunofluorescence detection (**e**) and statistical analysis (**f**) of TUNEL in heart tissues of WT or DR5-KO mice following sham or I40min/R24h. **g–h** mRNA expression of *Tnfa* and *Il1b* in the hearts of sham or I/R mice as detected by quantitative PCR (*n* = 3 for each group). **i–j** Representative results (**i**) and statistical analysis (**j**) of TTC-Evans blue double stain heart sections following I40min/R24h in WT mice treated with isotype antibody (*n* = 6), DR5-KO mice treated with isotype antibody (*n* = 6) and DR5-KO mice treated with anti-Ly6G antibody for neutrophils depletion (*n* = 5). Quantitative data are shown as means ± SEM. *P ≤ 0.05, **P ≤ 0.01, and ***P ≤ 0.001 as determined by two-tailed *t* test (**b**–**d**, **f**–**h**, and **j**)

Our previous studies have demonstrated that blocking the TRAIL-DR5 signaling pathway with sDR5-Fc can attenuate cardiac I/R injury in monkeys, pigs, and rats [14]. To further elucidate the underlying mechanisms, we validated this effect in DR5 knockout mice using the I40min/R24h model. DR5 knockout mice exhibited a reduced infarct area (Fig. 2a, b), decreased levels of CK (Fig. 2c) and LDH (Fig. 2d) in plasma, fewer TUNEL-positive cells in cardiac tissue (Fig. 2e, f), as well as lower transcription levels of *Il1b* and *Tnfa* in cardiac tissue detected by qPCR compared to the wild-type group (Fig. 2g, h). These results suggest that DR5 knockout can lessen cardiac IR injury in rodents.

We employed a well-established paradigm of anti-Ly6G-induced neutrophil depletion to investigate whether neutrophil-derived NETs have a detrimental role during the early inflammatory phase of cardiac electrical resistance injury. Neutrophil depletion resulted in a reduced infarct area compared to the control group. However, no significant differences in infarction area were found between the WT or DR5-KO groups after neutrophil depletion (Fig. 2i, j), suggesting that knocking out DR5 can reduce cardiac I/R injury, which is neutrophil dependent.

### Knockout of DR5 Reduces NETs Release During Cardiac IR Injury

**Fig. 3:**
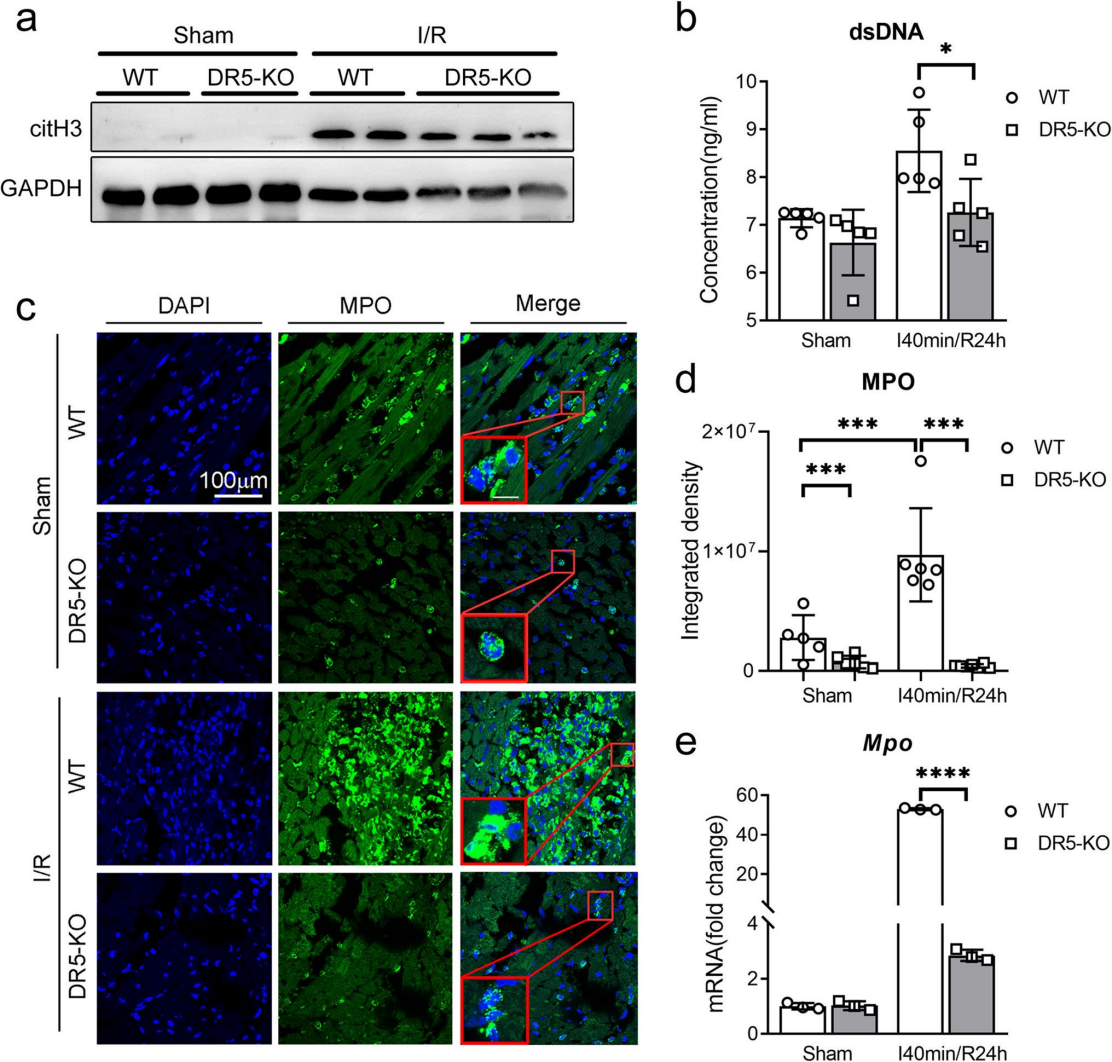
Knockout of DR5 decreases NETs after cardiac I/R injury. **a** The expression of citH3 in the hearts of WT or DR5-KO mice was assessed by western blot analysis following sham surgery or I40min/R24h. **b** Detection of dsDNA content in WT or DR5-KO mice plasma using ELISA assay following sham surgery or I40min/R24h. **c–d** Representative IF results (**c**) and statistical analysis (**d**) of MPO expression in WT or DR5-KO mice heart tissue following sham surgery or I40min/R24h.** e** mRNA expression of *Mpo* in WT or DR5-KO mice hearts following sham surgery or I40min/R24h as detected by quantitative PCR (*n* = 3 for each group). Quantitative data are shown as means ± SEM. *P ≤ 0.05, **P ≤ 0.01, and ***P ≤ 0.001 as determined by a two-tailed *t* test (**b**, **d**, and **e**)

NETs derived from neutrophils play an important role in the process of inflammation [18]. To investigate the impact of the TRAIL-DR5 pathway on NETs release in myocardial tissue and peripheral blood following cardiac IR injury, we collected the heart tissue and peripheral blood plasma. Western blot analysis revealed citH3 expression in all groups after I/R injury, with a higher level observed in the WT group compared to the DR5-KO group (Fig. 3a). ELISA analysis showed elevated levels of dsDNA content in plasma in the WT group and decreased dsDNA in DR5-KO mice after cardiac IR injury (Fig. 3b). Immunofluorescence results demonstrated a reduced presence of MPO-positive cells and citH3-positive cells in cardiac tissues (Fig. 3c, d). qRT-PCR analysis showed elevated *Mpo* mRNA levels in mouse heart tissue after I/R injury, with higher levels detected in the WT group compared to the DR5-KO group (Fig. 3e). These findings collectively imply that DR5 deletion can reduce the release of NETs from infiltrating neutrophils and peripheral blood.

### Blocking TRAIL-DR5 Pathway Through DR5-Fc Fusion Protein Reduces Heart NETs Release During Cardiac IR Injury

**Fig. 4:**
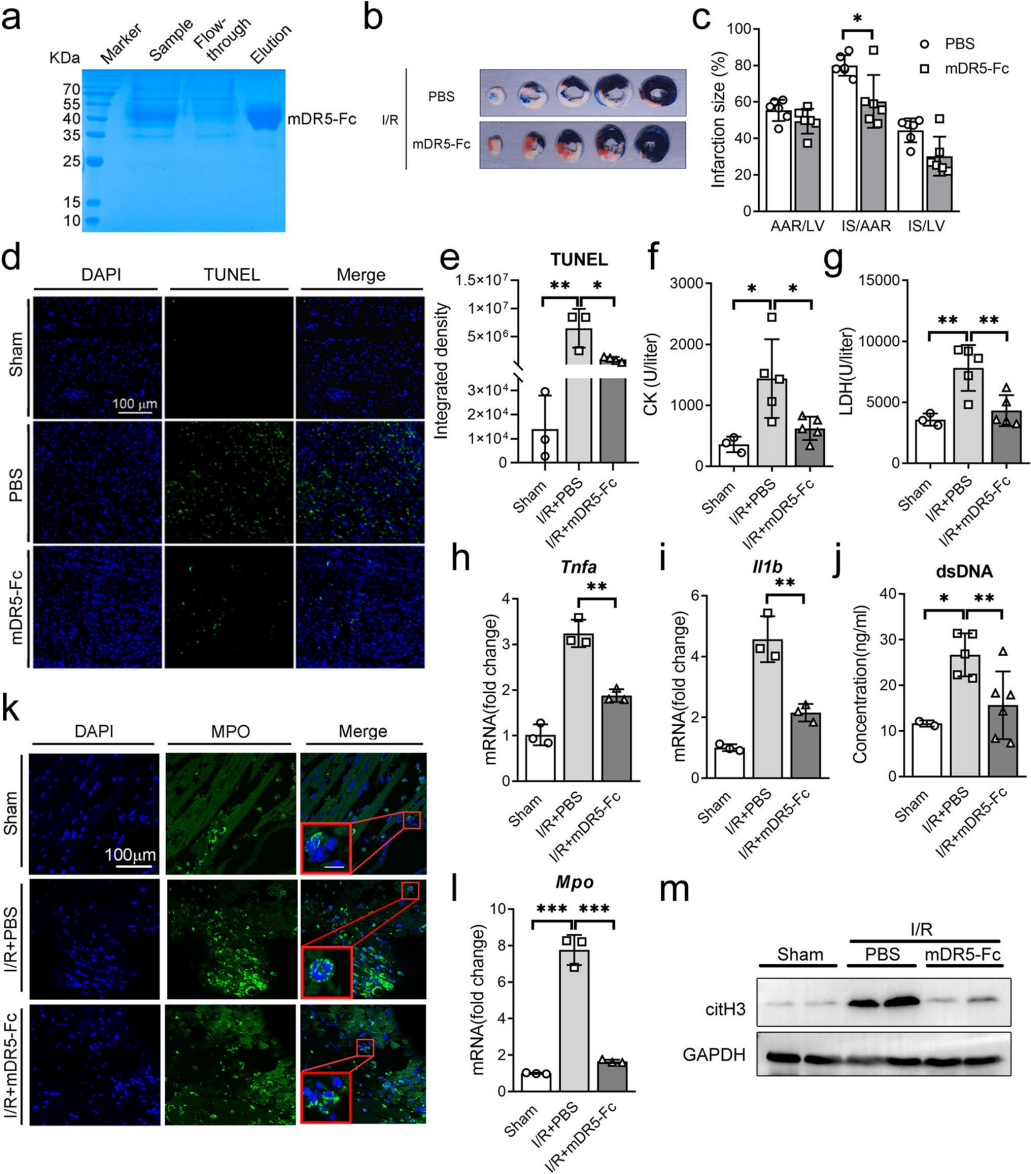
Administration of mDR5-Fc ameliorated mice’s cardiac I/R injury and attenuated the presence of NETs. **a** SDS-PAGE Coomassie brilliant blue staining for 293F culture media (sample), flow-through solution for sample affinity chromatography (flow-through), and purified mDR5-Fc (elution). **b–c** Representative results (**b**) and statistical analysis (**c**) of TTC-Evans blue double stain heart sections following sham or I40min/R24h in mice treated with PBS or mDR5-Fc. **d–e** Immunofluorescence detection (**d**) and statistical analysis (**e**) of TUNEL in heart tissues of mice following sham or I40min/R24h treated with PBS or mDR5-Fc.** f–g** Plasma concentrations of CK (**f**) and LDH (**g**) of mice following sham or I40minR/24h treated with PBS or mDR5-Fc were analyzed using the Catalyst Dx Chemistry Analyzer. **h–i** mRNA expression of *Tnfa* and *Il1b* in the hearts of sham or I40min/R24h mice treated with PBS or mDR5-Fc, as detected by quantitative PCR. **j** Detection of dsDNA content in mice plasma using ELISA assay following sham surgery or I40min/R24h treated with PBS or mDR5-Fc. **k** Immunofluorescence detection of MPO in mice heart tissue following sham surgery or I40min/R24h treated with PBS or mDR5-Fc. **l** mRNA expression of Mpo in the hearts of sham or I40min/R24h mice treated with PBS or mDR5-Fc, as detected by quantitative PCR. **m** The expression of citH3 in mice hearts was assessed by western blot analysis following sham or I40min/R24h treated with PBS or mDR5-Fc. Quantitative data are shown as means ± SEM. *P ≤ 0.05, **P ≤ 0.01, and ***P ≤ 0.001 as determined by a two-tailed *t* test (**c**) or one-way ANOVA followed by Tukey’s post hoc test (**e**–**j**, and **l**)

The efficacious blocking of the TRAIL-DR5 signaling pathway and the cardioprotective impact of the human sDR5-Fc antibody fusion protein in rats, pigs, and monkeys have been verified by our earlier investigation. However, given the modest homology of approximately 63% between mouse and human TRAIL, we developed a mouse-derived mDR5-Fc antibody fusion protein using the 293F protein eukaryotic expression system and purified it with affinity chromatography. mDR5-Fc was identified through Coomassie brilliant blue staining (Fig. 4a). Twenty-four hours after cardiac I/R injury, we observed a significant decrease in the area of myocardial infarction (Fig. 4b, c), less TUNEL positive in mouse heart tissue (Fig. 4d, e), reduced levels of plasma CK (Fig. 4f) and LDH (Fig. 4g), as well as lower transcription levels of *Tnfa* (Fig. 4h) and *Il1b* (Fig. 4i) in cardiac tissue detected by qPCR compared to the wild-type group in mice treated with mDR5-Fc compared to the PBS group. According to these findings, mice’s myocardial I/R damage is well mitigated by the mDR5-Fc fusion protein.

Considering the reduced release of NETs observed in DR5 knockout mice following I/R injury, we investigate the potential impact of mDR5-Fc on NETs release. Higher levels of dsDNA in plasma were observed in mice treated with PBS than those treated with mDR5-Fc (Fig. 4j). Moreover, immunofluorescence results revealed a notable decrease of MPO-positive cells within cardiac tissues subsequent to mDR5-Fc therapy (Fig. 4k). qRT-PCR analysis revealed significantly increased mRNA levels of *Mpo* in mouse cardiac tissue of the PBS group following I/R injury compared to the mDR5-Fc group (Fig. 4l). Western blot analysis of mouse cardiac tissue revealed an upregulation in the expression of citH3 following I/R injury compared to the sham group, with significantly higher levels observed in mice treated with PBS as opposed to those treated with mDR5-Fc (Fig. 4m). These results imply that mDR5-Fc treatment confers protection against myocardial I/R injury by efficiently reducing the release of NETs from cardiac tissues.

### Blocking TRAIL-DR5 Pathway Reduces Neutrophil Infiltration by Diminishing Chemokines and Adhesion Molecules

**Fig. 5:**
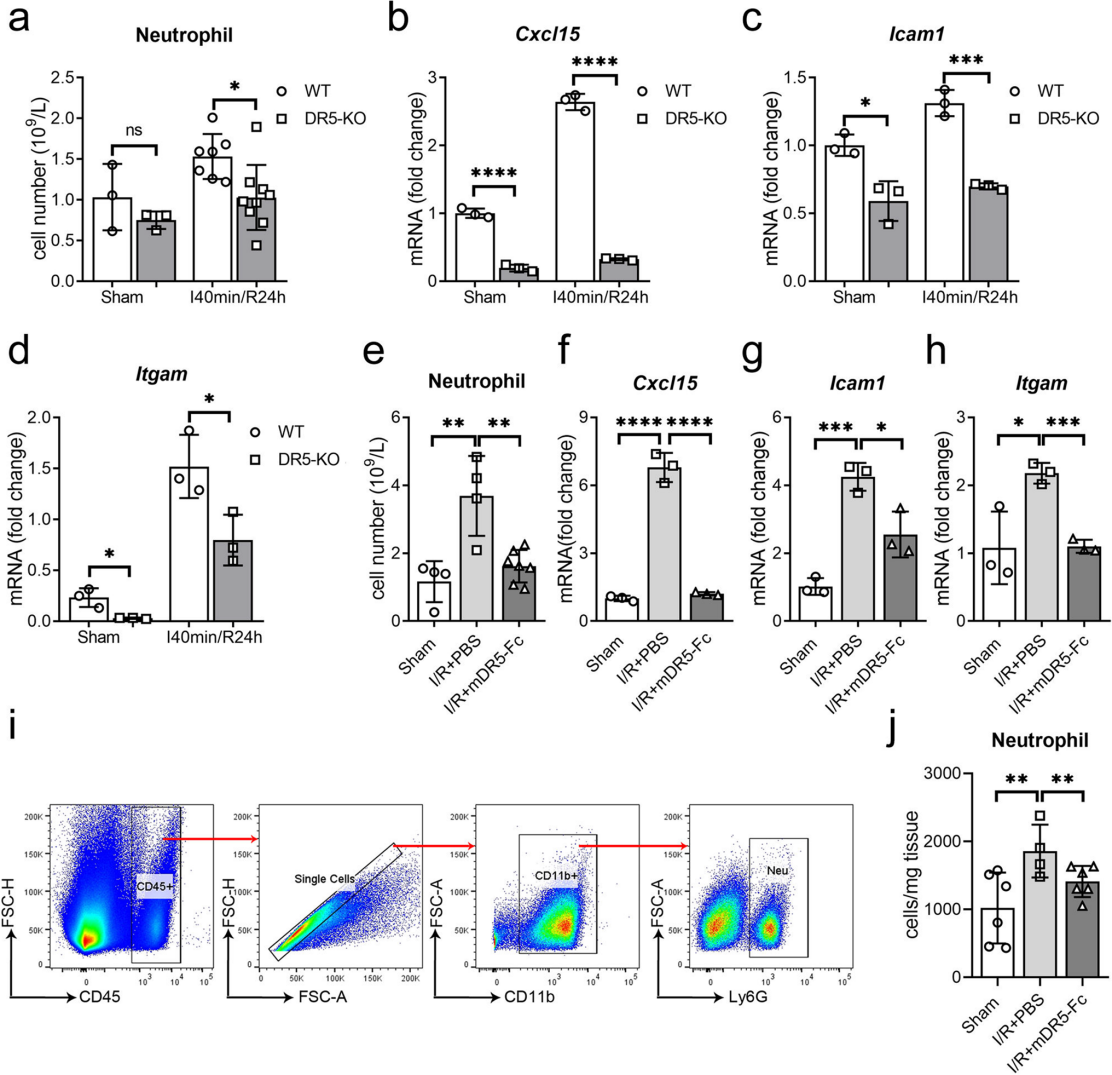
Blocking the TRAIL-DR5 pathway reduces NEU infiltration. **a** Quantification of neutrophils in the peripheral blood of WT or DR5-KO mice following sham surgery or I40min/R24h, as detected by Auto Hematology Analyzer. **b–d** mRNA expression of *Cxcl15, Icam1 and Itgam* in the WT or DR5-KO mice hearts following sham or I40min/R24h treatment, as detected by quantitative PCR. **e** Quantification of neutrophils in the peripheral blood of mice following sham surgery or I40min/R24h treated with PBS or mDR5-Fc, as detected by Auto Hematology Analyzer. **f–h** mRNA expression of *Cxcl15, Icam1, and Itgam* in mice hearts following sham surgery or I40min/R24h treated with PBS or mDR5-Fc, as detected by quantitative PCR. **i** Gating strategy for neutrophils analysis in mice cardiac tissue using flow cytometry. **j** Quantitative analysis of neutrophil infiltration in mice heart tissue, the data was quantified by neutrophils numbers per milligram of cardiac tissue. Quantitative data are shown as means ± SEM. *P ≤ 0.05, **P ≤ 0.01, and ***P ≤ 0.001 as determined by a two-tailed *t* test (a–d) or one-way ANOVA followed by Tukey’s post hoc test (**e**–**h** and **j**)

Neutrophils respond to adhesion and chemotaxis, infiltrating the damaged tissue and releasing NETs, cytokines, and chemokines to exacerbate the inflammatory [19–21] response further. To investigate whether the reduction of NETs in the cardiac tissue after I/R injury in DR5 knockout mice and mDR5-Fc treated mice is attributed to a decline in neutrophil population, we quantified the neutrophil count in both peripheral blood and cardiac tissue while also evaluating the expression levels of chemotactic, adhesion, and integrin molecules within the cardiac tissue. The blood routine tests revealed a significant reduction in the neutrophil count in the peripheral blood of both DR5-KO and mDR5-Fc treated groups following cardiac I/R (Fig. 5a, e). Additionally, qPCR analysis revealed a significant downregulation of key adhesion molecule *Icam1*, chemokine *Cxcl15*, and integrin *Itgam* expression levels in DR5 knockout mouse hearts compared to WT controls (Fig. 5b–d). Similarly, treatment with mDR5-Fc resulted in reduced expression levels of *Icam1*, *Cxcl15*, and *Itgam* compared to mouse hearts treated with PBS (Fig. 5f–h). Flow cytometry results revealed a significant reduction in infiltrating neutrophils within the cardiac tissue of mDR5-Fc-treated mice 24 h post-I/R (Fig. 5i, j). According to these results, blocking the TRAIL-DR5 signaling pathway may be a useful strategy to lessen neutrophil infiltration into I/R hearts and help lower the number of NETs produced.

### Blocking TRAIL-DR5 Pathway Through mDR5-Fc Administration Directly Attenuates NETs Release

**Fig. 6:**
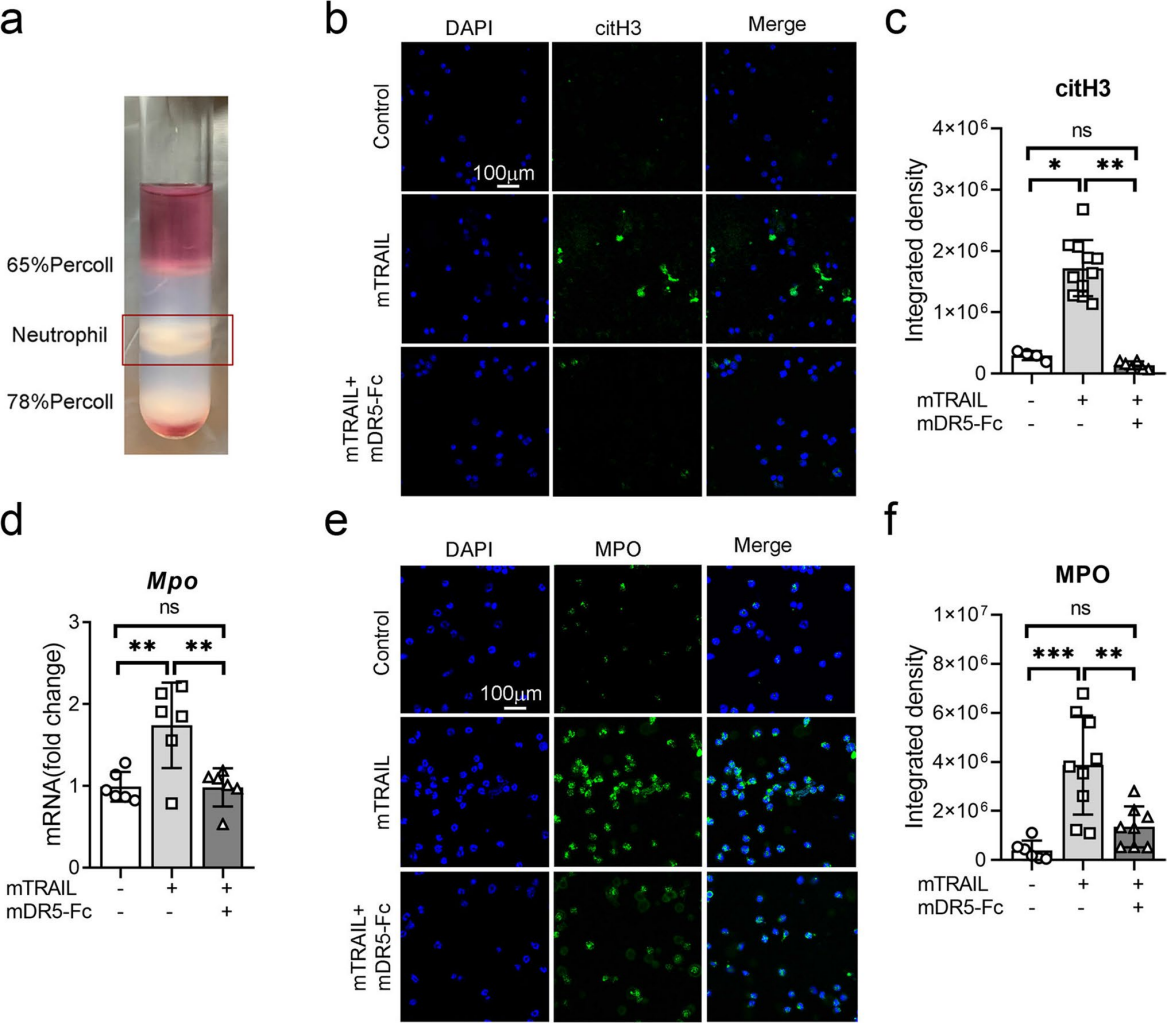
Administration of mDR5-Fc directly attenuates NETs release of bone marrow-derived neutrophil. **a** Percoll density gradient centrifugation was used for the isolation of neutrophils derived from bone marrow. **b–c** Immunofluorescence detection (**b**) and statistical analysis (**c**) of citH3 in neutrophils treated with PBS (control), mTRAIL alone, or co-administration of mTRAIL and mDR5-Fc. **d** mRNA expression of *Mpo* in neutrophils treated with or without mTRAIL or co-administration of mTRAIL and mDR5-Fc, as detected by quantitative PCR. **e–f** Immunofluorescence detection (**e**) and statistical analysis (**f**) of MPO in neutrophils treated with PBS (control), mTRAIL alone, or co-administration of mTRAIL and mDR5-Fc. Quantitative data are shown as means ± SEM. *P ≤ 0.05, **P ≤ 0.01, and ***P ≤ 0.001 as determined by one-way ANOVA followed by Tukey’s post hoc test (**c**, **d**, and **f**)

We used Percoll density gradient centrifugation to separate bone marrow-derived neutrophils to analyze the direct effect of blocking the TRAIL-DR5 signaling pathway on NETs release (Fig. 6a). The isolated neutrophils were exposed to mouse TRAIL recombinant protein (mTRAIL) and/or mDR5-Fc, resulting in a significant upregulation of citH3 expression as detected by immunofluorescence (Fig. 6b–c). The administration of mDR5-Fc resulted in a significant reduction in *Mpo* compared to the mTRAIL-treated group, as detected by qPCR and immunofluorescence staining (Fig. 6d–f). Accordingly, these findings suggest that the activation of the DR5 signaling pathway directly triggers neutrophil formation of NETs, while mDR5-Fc inhibits NETs induced by mTRAIL.

### Administration of mDR5-Fc Inhibits the Formation of NETs, Thereby Attenuating Apoptosis in Cardiomyocytes

**Fig. 7:**
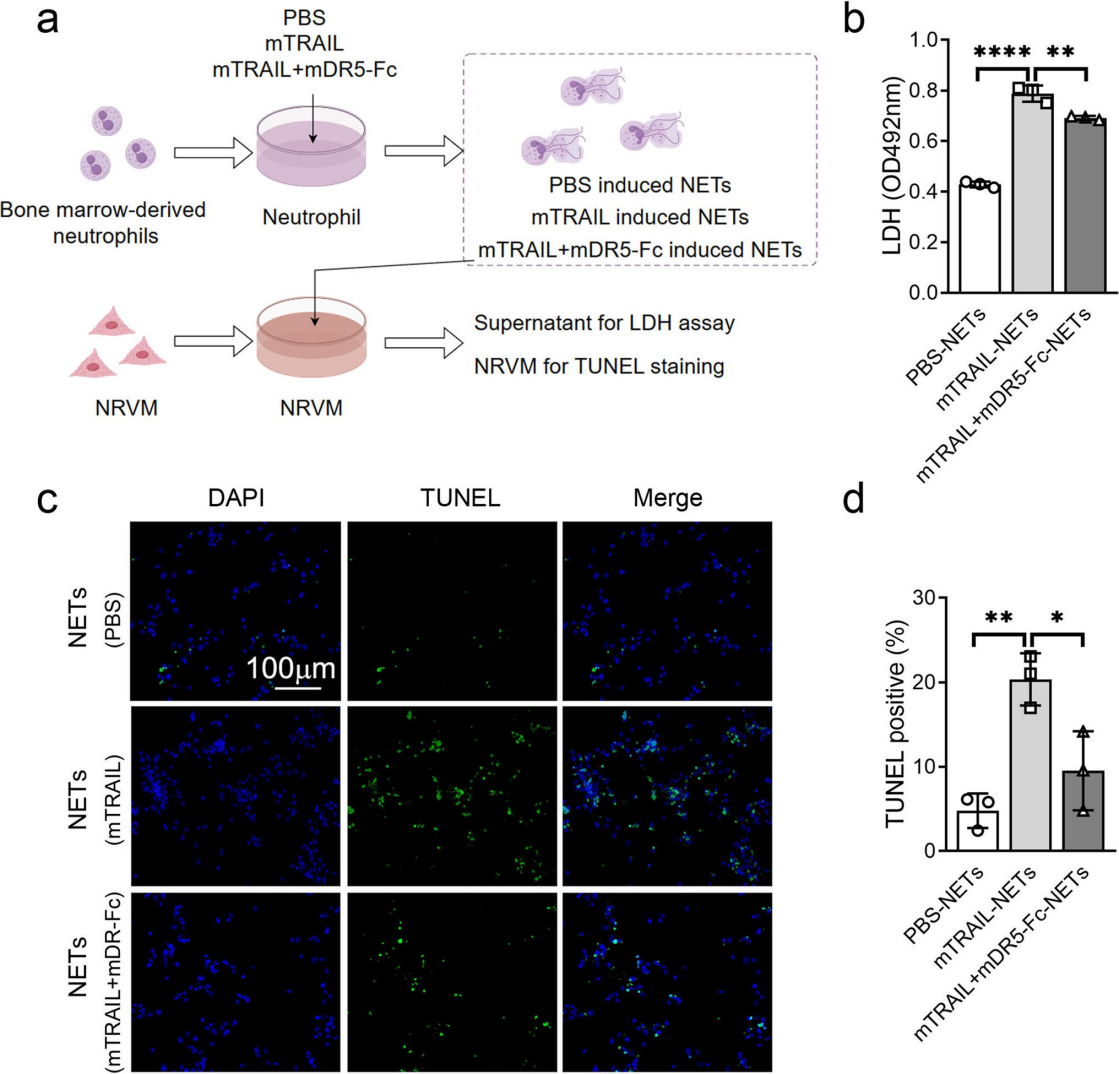
The administration of mDR5-Fc effectively attenuates the detrimental effects of NETs induced by sTRAIL, thus protecting NRVM.** a** A diagram for the induction of NETs and treatment of NRVM (by Figdraw). **b** Detection of LDH expression in the culture supernatant of NRVMs induced by NETs obtained from various treatments. **c–d** Immunofluorescence detection (**c**) and statistical analysis (**d**) of TUNEL in NRVM treated with NETs obtained from various treatments. Quantitative data are shown as means ± SEM. *P ≤ 0.05, **P ≤ 0.01, and ***P ≤ 0.001 as determined by one-way ANOVA followed by Tukey’s post hoc test (**b** and** d**)

NETs are essential in the pathophysiology of cardiovascular disorders because they can cause harm to cardiac cells. To investigate the potential protective effect of mDR5-Fc against myocardial tissue damage induced by NET formation, we conducted a co-culture experiment to assess the impact of neutrophil-derived NETs on NRVM apoptosis. After co-culturing with pre-treated neutrophils-derived NETs, NRVM was tested for TUNEL staining. The supernatant was collected for LDH assay (Fig. 7a). Increased LDH was observed in NRVMs culture media, wherein the mTRAIL-induced NETs stimulated group exhibited significantly higher levels compared to the mDR5-Fc blockade group (Fig. 7b). Similarly, immunofluorescence-based TUNEL detection revealed enhanced apoptosis in NRVM cells induced by mTRAIL-induced NETs. In contrast, less apoptosis was detected in NRVM treated with mTRAIL/mDR5-Fc induced NETs (Fig. 7c–d). These results imply that myocardial damage, which can be effectively mitigated by mDR5-Fc, may be exacerbated by the induction of NETs by mTRAIL.

### Blocking the TRAIL-DR5 Signaling Pathway Can Effectively Downregulate PAD4 Expression

**Fig. 8:**
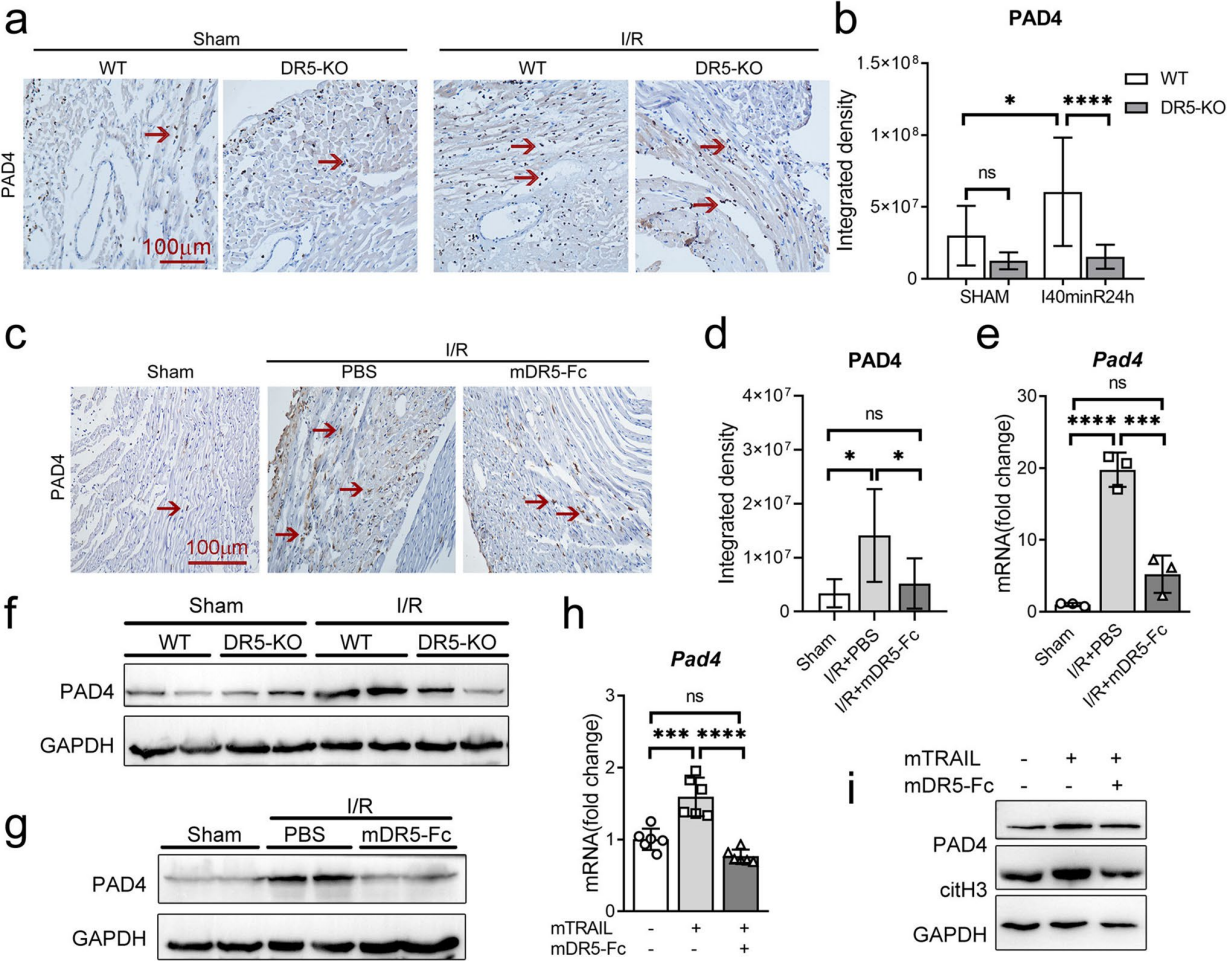
Blocking the TRAIL-DR5 pathway could suppress PAD4 expression. **a–b** Representative IHC results (**a**) and statistical analysis (**b**) of PAD4 expression in WT or DR5-KO mice heart tissue following sham surgery or I40min/R24h. **c–d** Representative IHC results (**c**) and statistical analysis (**d**) of PAD4 expression in mouse heart tissue following sham surgery or I40min/R24h treated with PBS or mDR5-Fc. **e** The expression of *Pad4* in mouse heart tissue following sham surgery or I40min/R24h treated with PBS or mDR5-Fc, as detected by quantitative PCR. **f** The expression of PAD4 in WT or DR5-KO mice hearts was assessed by western blot analysis following sham or I40min/R24h. **g** The expression of PAD4 in mouse heart tissue following sham surgery or I40min/R24h treated with PBS or mDR5-Fc, as detected by western blot. **h–i** The expression of PAD4 in bone marrow-derived neutrophils treated with PBS, mTRAIL alone, or co-administration of mTRAIL and mDR5-Fc, as detected by quantitative PCR (**h**) and western blot (**i**). Quantitative data are shown as means ± SEM. *P ≤ 0.05, **P ≤ 0.01, and ***P ≤ 0.001 as determined by a two-tailed *t* test (**b**) or one-way ANOVA followed by Tukey’s post hoc test (**d**, **g**, and **i**)

Peptidyl arginine deiminase 4 (PAD4) is a pivotal histone-modifying enzyme implicated in the mediation of NETs formation following ischemia/reperfusion injury [22, 23]. To elucidate the inhibitory mechanism of NETs release by blocking the TRAIL-DR5 pathway, we evaluated the impact of TRAIL-DR5 on PAD4 expression. Immunohistochemistry analysis revealed an upregulation of PAD4 expression in the hearts of mice subjected to IR injury, whereas knockout of DR5 (Fig. 8a–b) or administration of mDR5-Fc (Fig. 8c–d) resulted in a substantial reduction in PAD4 expression. qPCR analyses revealed an upregulation of *Pad4* expression in the mouse heart following ischemia/reperfusion (I/R) injury, which could be effectively attenuated by administration of mDR5-Fc (Fig. 8e). Additionally, western blot analyses revealed an upregulation of PAD4 expression in the mouse heart following ischemia/reperfusion (I/R) injury, which could be effectively attenuated by knocking out DR5 or administration of mDR5-Fc (Fig. 8f–g). Moreover, qPCR and western blot analyses demonstrated an upregulation of PAD4 expression in bone marrow-derived neutrophils treated with mTRAIL. However, this effect was attenuated upon treatment with mDR5-Fc (Fig. 8h–i). These results imply that blocking the TRAIL-DR5 pathway reduces the expression of PAD4, which in turn reduces the release of NETs.

## Discussion

Heart I/R injury is a serious clinical issue that needs to be addressed right away since it involves complex pathogenic mechanisms such as an abrupt increase in reactive oxygen species, calcium overload, and an inflammatory response during vascular reperfusion. Currently, there is a lack of specific drug prevention and treatment strategies targeting heart I/R injury and therapeutic targets. Owing to the complexity of the underlying process causing this illness, several studies have been conducted looking at different ways to reduce reperfusion injury from different angles. Inflammatory response plays a pivotal role in acute myocardial infarction and I/R injury, leading researchers to explore multiple endeavors aimed at mitigating the inflammatory response [24–27]. For instance, studies have revealed that S100a8/a9 levels rapidly increase during the early stages of cardiac I/R injury [28]. Knocking out S100A9 or employing neutralizing antibodies against S100A9 alleviates cardiac I/R injury in mice [29]. Dectin-1 expressed by macrophages exacerbates myocardial I/R injury by regulating macrophage polarization and recruiting neutrophil infiltration, thus making it a potential target for cardiac therapy [30]. The clinical trials have utilized Anakinra, an Il-1β receptor antagonist [31–33], and etanercept, a TNF-α antibody [34], to inhibit inflammatory mediators that exacerbate myocardial I/R injury.

In our previous study, we discovered that inhibition of the TRAIL-DR5 signaling pathway could attenuate myocardial cell death and mitigate inflammatory responses, thereby reducing infarct size and ameliorating heart I/R injury. We found that monocytes/macrophages and granulocytes are required for the protective effects of sDR5-Fc [14]. Blocking the TRAIL-DR5 pathway modifies neutrophil involvement in cardiac I/R damage, although the mechanism behind this modulation remains unknown.

Through NETosis, neutrophils can release NETs that aggregate platelets and encourage thrombus formation, aggravating inflammation and inducing an innate immune response [35–37] that can lead to heart damage. However, the role of neutrophils in cardiac injury remains multifaceted. Some studies have demonstrated that inhibiting neutrophils can ameliorate acute inflammation in a mouse model of viral myocarditis [38]. Previous studies have demonstrated that depletion of neutrophils can attenuate infarct size in a mouse model of myocardial I/R, which is consistent with our research findings. However, it has been observed that neutrophil depletion does not impact infarct size within 24 h after myocardial infarction but progressively impairs cardiac function in a mouse model of permanent LAD occlusion. Neutrophils are capable of promoting macrophage polarization towards the M2 phenotype during cardiac repair and remodeling processes, highlighting their beneficial contribution [39]. These differences could be the result of differences in treatment regimens, animal models, and detection intervals. Our findings reveal that employing Ly6G antibodies for neutrophil removal leads to a reduction in I/R-induced myocardial infarct area at 24 h post-injury, indicating that early neutrophil suppression may be advantageous for cardioprotection in cases of I/R damage.

The activation of the TRAIL-DR5 pathway can induce apoptosis through death-inducing signaling complex (DISC) formation and subsequent activation of caspase 3, or trigger an inflammatory response via activation of the NF-κB transcription factor [40, 41]. In the context of acute myocardial infarction and cardiac I/R injury, cardiomyocytes undergo cell death and release damage-associated molecular patterns (DAMPs), which activate inflammation and upregulate chemokines and adhesion molecules, thereby promoting neutrophil infiltration. Therefore, we looked into how blocking the TRAIL-DR5 pathway affected the invasion of neutrophils. By deleting DR5 or administering mDR5-Fc, we successfully ameliorated cardiac reperfusion injury, suppressed the gene expression of pro-inflammatory factors *Il1b* and *Tnfa*, chemokine *Cxcl15*, as well as adhesion molecule *Vcam1*, additionally reducing neutrophil infiltration in the heart. Previous studies have demonstrated that NETosis primarily requires PAD4 in response to calcium ionophores and immune complexes involving chromatin decondensation and nuclear envelope rupture [11, 42]. We observed that inhibition of the TRAIL-DR5 pathway could suppress PAD4 expression in NEU cells and cardiac tissue. As a result, DR5 deletion or mDR5-Fc administration efficiently prevented the creation of NETs via a variety of methods.

We studied the pharmacodynamics of sDR5-Fc in rhesus monkeys, pigs, and rats as animal models. However, owing to the unavailability of antibodies for mechanistic research purposes and the high cost involved in conducting such studies using large animal models, we opted to perform mechanistic research using mice instead. Initially, we validated our findings using DR5 knockout mice and observed that knocking out DR5 can alleviate cardiac I/R injury. On the other hand, in a mouse cardiac I/R model, the protective impact of human-derived sDR5-Fc was insufficient. The subsequent investigation revealed a species disparity in mouse DR5, with a 38.61% homology to humans, and in mouse TRAIL, exhibiting a 63.35% homology to humans (as predicted by UniProt). Additionally, it was found that while the extracellular domain of mouse DR5 possesses multiple N-glycosylation sites, the human-derived DR5 extracellular domain lacks any N-glycosylation sites [43, 44]. This discrepancy partly explains the low effectiveness of human-derived sDR5-Fc in the mouse cardiac IR model. As a result, we created mouse-derived mDR5-Fc for additional mechanistic studies.

**Fig. 9:**
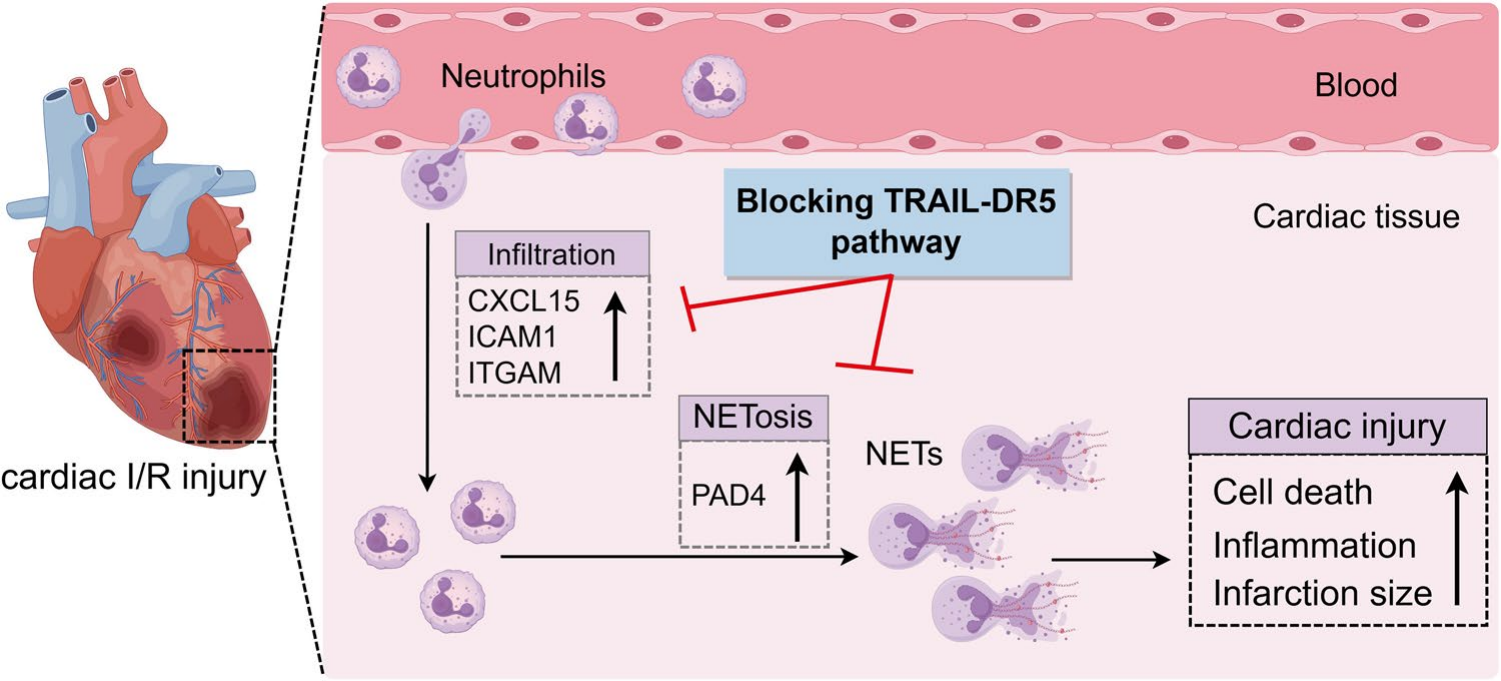
A summary diagram illustrates the impact of blocking the TRAIL-DR5 pathway on neutrophil infiltration and NETs formation following cardiac I/R injury (by Figdraw). In a mouse model of cardiac ischemia for 40 min followed by reperfusion for 24 h, inhibition of the TRAIL-DR5 signaling pathway resulted in decreased expression of the chemokine CXCL15, adhesion molecule ICAM1, and integrin ITGAM, thereby attenuating neutrophil infiltration. Simultaneously, blockade of the TRAIL-DR5 signaling pathway directly suppressed PAD4 expression in neutrophils, leading to reduced formation of NETs. These effects collectively contribute to diminished cardiomyocyte death, alleviation of the cardiac inflammatory response, and ultimately result in a reduced myocardial infarct size

In this study, we show that by lowering the development of NETs in the heart, suppression of the TRAIL-DR5 signaling pathway attenuates myocardial I/R injury. Neutrophils play a pivotal role in this process, and blockade of TRAIL-DR5 not only diminishes adhesion and chemotactic factors to decrease neutrophil infiltration but also directly suppresses PAD4 expression to inhibit NETs formation. Moreover, our findings suggest that TRAIL-induced NETs release promotes primary cardiomyocyte apoptosis, which can be blocked by mDR5-Fc (Fig. 9). Although neutrophils are detrimental during the acute phase of myocardial infarction, careful consideration is necessary due to their beneficial role in the healing phase. These dual effects, like a “double-edged sword,” may present challenges in clinical translation. Therefore, further comprehensive research is urgently needed to investigate aspects such as optimal timing and dosage of administration, as well as underlying mechanisms. On the basis of essential mechanistic studies, sDR5-Fc may be explored in clinical settings as a potential treatment drug for heart IR injury.

## Data Availability

The data that support the findings of this study are available from the corresponding author upon reasonable request.
